# In Silico Evaluation of Algorithm-Based Clinical Decision Support Systems: Protocol for a Scoping Review

**DOI:** 10.2196/63875

**Published:** 2025-01-16

**Authors:** Michael Dorosan, Ya-Lin Chen, Qingyuan Zhuang, Shao Wei Sean Lam

**Affiliations:** 1 Health Services Research Centre Singapore Health Services Pte Ltd Singapore Singapore; 2 Department of Biomedical Informatics and Medical Education University of Washington Seattle, WA United States; 3 Division of Supportive and Palliative Care National Cancer Centre Singapore Singapore Singapore; 4 Data and Computational Science Core National Cancer Centre Singapore Singapore Singapore; 5 Duke-NUS Medical School National University of Singapore Singapore Singapore; 6 Health Services Research Institute SingHealth Duke-NUS Academic Medical Centre Singapore Singapore; 7 Health Services and Systems Research Duke-NUS Medical School National University of Singapore Singapore Singapore; 8 Lee Kong Chian School of Business Singapore Management University Singapore Singapore

**Keywords:** clinical decision support algorithms, in silico evaluation, clinical workflow simulation, health care modeling, digital twin, quadruple aims, clinical decision, decision-making, decision support, workflow, support system, protocol, scoping review, algorithm-based, screening, thematic analysis, descriptive analysis, clinical decision-making

## Abstract

**Background:**

Integrating algorithm-based clinical decision support (CDS) systems poses significant challenges in evaluating their actual clinical value. Such CDS systems are traditionally assessed via controlled but resource-intensive clinical trials.

**Objective:**

This paper presents a review protocol for preimplementation in silico evaluation methods to enable broadened impact analysis under simulated environments before clinical trials.

**Methods:**

We propose a scoping review protocol that follows an enhanced Arksey and O’Malley framework and PRISMA-ScR (Preferred Reporting Items for Systematic Reviews and Meta-Analyses Extension for Scoping Reviews) guidelines to investigate the scope and research gaps in the in silico evaluation of algorithm-based CDS models—specifically CDS decision-making end points and objectives, evaluation metrics used, and simulation paradigms used to assess potential impacts. The databases searched are PubMed, Embase, CINAHL, PsycINFO, Cochrane, IEEEXplore, Web of Science, and arXiv. A 2-stage screening process identified pertinent articles. The information extracted from articles was iteratively refined. The review will use thematic, trend, and descriptive analyses to meet scoping aims.

**Results:**

We conducted an automated search of the databases above in May 2023, with most title and abstract screenings completed by November 2023 and full-text screening extended from December 2023 to May 2024. Concurrent charting and full-text analysis were carried out, with the final analysis and manuscript preparation set for completion in July 2024. Publication of the review results is targeted from July 2024 to February 2025. As of April 2024, a total of 21 articles have been selected following a 2-stage screening process; these will proceed to data extraction and analysis.

**Conclusions:**

We refined our data extraction strategy through a collaborative, multidisciplinary approach, planning to analyze results using thematic analyses to identify approaches to in silico evaluation. Anticipated findings aim to contribute to developing a unified in silico evaluation framework adaptable to various clinical workflows, detailing clinical decision-making characteristics, impact measures, and reusability of methods. The study’s findings will be published and presented in forums combining artificial intelligence and machine learning, clinical decision-making, and health technology impact analysis. Ultimately, we aim to bridge the development-deployment gap through in silico evaluation-based potential impact assessments.

**International Registered Report Identifier (IRRID):**

DERR1-10.2196/63875

## Introduction

The recent advent of artificial intelligence (AI) in clinical decision support (CDS) systems furthers the intended purpose of such systems to enhance medical decision-making by using clinical knowledge and other health information [1]. Traditionally, such systems rely on either standard of care or knowledge-based models [2]. AI models’ statistical learning capability—iteratively identifying and learning patterns from large volumes of data—facilitates including more information to arrive at an optimal decision recommendation [3,4]. Despite the prevalence of AI-based CDS development [5], adoption has been limited [6,7]. A significant barrier to adoption is the high trust and safety requirement of health care applications that demand evidence on implications to the broader system and the clinical workflows across the care value stream [5,8,9].

The current state of reporting traditional statistical analysis and CDS development predominantly focuses on accuracy, sensitivity, specificity, goodness-of-fit, and other discrimination-related measures [3] that do not precisely measure patient-, provider-, process-, and cost-related outcomes. These metrics may not capture the actual clinical improvements or the broader consequences that may arise when implementing CDS within the clinical workflows. For example, such metrics may be limited or require calibration when considering context-specific tradeoffs in predictive accuracies. Preference may be higher sensitivity than specificity in different contexts, such as early screening. Conversely, an oversensitive screen may result in more resources needed for confirmatory testing [10]. Hence, the ability to simulate the impact of model calibration and thresholding decisions for downstream workflows and eventual outcomes may be beneficial, especially when provider and process resources could render any CDS-based recommendation impracticable.

Traditional assessments of the implications of health technologies, such as algorithm-based CDS, focus on generating clinical evidence through randomized controlled trials to balance assessment scope and feasibility [11]. However, such assessments are challenged by the following: (1) the need for continuous evaluation of impact both in the development stage [12,13] and when such systems are deployed [14,15], (2) the need for more robust evidence that accounts for variations among real-world care pathways—characterized by heterogeneous settings and patient populations [5,9,16,17], and (3) the cost of an expanded scope of assessment when considering a broader health care pathway or system [18] such as in the evaluation of bundled payments in health care pathways and value-based health care [19,20].

In silico methods that simulate real-world care pathways present an alternative to evaluate CDS at preimplementation while approximating real-world care pathway events, behaviors, disease states, and resource constraints. These methods enable an iterative analysis of different clinical workflow scenarios, addressing the need for continuous impact evaluation without significant resource demands and disruptions to ongoing usual care practice [11,21,22]. In addition, such methods allow for the integral consideration of practical constraints [11,23], such as when at-risk patients are correctly identified by a CDS but cannot receive the appropriate downstream interventions due to resource constraints [24,25]. The value of clinical workflow simulations as an alternative is put forward by the recent inclusion of preclinical evaluation of CDS in guidelines for academic research reporting. Specific to AI-based CDS, the development and exploratory clinical investigations of decision support systems driven by AI reporting guidelines strongly endorse the concept of preclinical or in silico evaluation—that is, via computer simulations before the first clinical trial [18]. Vasey et al [21] cite the development of other guidelines, for example, transparent reporting of a multivariable prediction model for individual prognosis or diagnosis–artificial intelligence [26,27] and standards for reporting of diagnostic accuracy study–artificial intelligence [28], that tackle the reporting of AI-based CDS evaluation at the preclinical stage. In addition, in silico modeling has been previously argued to be beneficial in drug discovery [29], surgical systems innovation [30], and biomedical product regulation [10], as it can address the trade-off between scope and resource demands existing in traditional impact assessments.

Evaluating CDS under uncertainty can leverage mathematical models that consider the inherent stochasticity of clinical workflows and systems, such as simulation models [22]. As decisions are often time-sensitive [31], simulations should also be dynamic. Discrete events simulation (DES) and agent-based models (ABM) are stochastic dynamic models frequently used [32]. These simulation models allow context-specific domain nuances to be captured in the model logic as discrete states, actions, and transitions, thereby capturing the unique characteristics and uncertainties that define clinical workflows across care settings and sites. Queueing, Markov, and other stochastic process models [22,33,34] are closely associated with these models. System-level workflow simulations such as those using DES [22] and ABM [35] can model intricate health care dynamics and are commonly applied to model clinical workflows in health service delivery. These methods facilitate comprehensive analyses encompassing patient journey, resource use, and stakeholder interactions, providing insights into operational efficiencies, bottlenecks, and unintended consequences of implementing automated decision-support interventions. It offers a flexible method to capture the dynamic workflows in which the entities (ie, patients and providers) interact and are exposed to important clinical/process events (ie, admission and clinical decision) while consuming and releasing resources (ie, number of nurses in a hospital ward at a given time interval) [36]. By adjusting the decision thresholds, we can evaluate the CDS’ impact using a variety of decision-analytic measures, for example, decision curve analysis [37] to determine the most clinically helpful prediction model.

Given the significance of such in silico evaluation via simulation models, a consolidated knowledge base will help to guide their use in evaluating CDS systems. Current research needs to be more cohesive, with disparate methodologies focusing on narrow facets of health care delivery [38,39]. To support the advancement of workflow-sensitive evaluation methodologies for CDS systems, we propose a scoping review protocol that investigates the following components of in silico evaluation: (1) the use of more pragmatic measures of impact that are relevant to the quadruple aims of health care [40,41], and (2) the simulation modeling paradigm used. Specifically, we propose a review protocol that (1) maps out the state-of-art development and application of in silico clinical workflows to evaluate algorithm-based CDS—both traditional statistical analysis- and AI-based—models and (2) identifies relevant research gaps. To our knowledge, this is the first scoping review on in silico evaluation strategies for AI applications in CDS using workflow simulation methods.

## Methods

### Overview

We followed the stages in a scoping review proposed in the Arksey and O’Malley framework [42] while considering more recent enhancements [43-45] for each stage. Specifically, we followed the steps of (1) identifying the research question, (2) searching and identifying relevant studies, (3) study selection, (4) data extraction, (5) collection, summarizing, and reporting of findings, and (6) consultation with stakeholders. These are detailed in the succeeding sections.

### Stage 1: Identifying the Research Question

This scoping review endeavors to synthesize existing knowledge on the in silico evaluation of algorithm-based CDS systems via clinical workflow simulation methods. High-fidelity workflow simulations offer a pragmatic solution by allowing in silico replication of clinical processes, predicting the behavior of systems, and assessing the potential impacts of new models without risking patient safety or disrupting existing services [46]. To identify the scope of the review, we first conducted a rapid scan [45] of existing academic articles that discuss the evaluation of algorithm-based CDS. This review includes those using standards of care, knowledge bases, or AI to support a clinical decision recommendation [2]. In succeeding sections, these are generically referred to as CDS.

Regular team discussions were conducted to summarize findings and shortlist authors who publish peer-reviewed journal articles in our field of interest. Our study team comprises researchers with collective experience in machine learning model development, conducting systematic review studies, systems optimization research, and medical research and practice. The authors of this protocol—a senior clinician (QZ), a senior data scientist (SSWL), a junior data scientist (MD), and a junior pharmacoepidemiology researcher (YLC)—serve as the initial review team. After several initial iterations, we agreed on the research questions in Textbox 1. Further, we identified a list of concepts and accompanying keywords relevant to our main research question. These are presented in Table 1. Our focus lies in the exploration of simulation methods, particularly their application to clinical decision-making tasks. We aim to examine how these simulation models are developed, implemented, and evaluated. Additionally, we seek to identify gaps within the existing body of literature, specifically concerning the design and assessment of simulation-based approaches in health care.

**Main and specific research questions.**

**Main research question (RQ):**

- What are the proposed in silico potential impact evaluation strategies for clinical decision support (CDS) systems?

**Specific RQs:**

- RQ1: What are the reported clinical decision tasks and domains that report the use of CDS?
- RQ2: What metrics are reportedly used for evaluating potential impact?
- RQ3: What simulation modeling paradigms are used?
- RQ4: What are the intended objectives of the simulation modeling frameworks used?
- RQ5: What are the gaps in existing literature of in silico CDS evaluation?

**Table 1:** Concept framework used in searching relevant articles.

Key concepts	Keywords
Clinical decision support models, algorithms, and systems	Machine learning, deep learning, artificial intelligence, reinforcement learning, supervised machine learning, unsupervised machine learning, semisupervised machine learning, self-supervised machine learning, expert system
Objective of the CDS^a^ model	Clinical decision support, clinical decision-making, prognosis, diagnosis, screening, triage
Evaluation objective	Validation, potential impact, impact assessment, decision analysis, decision analytics measure, model calibration, model tuning, credibility, cost-benefit analysis
Evaluation strategy	In silico, computer simulation, digital twin, simulation, preimplementation, predeployment, computational simulation

^a^CDS: clinical decision support.

### Stage 2: Identifying Relevant Studies

In identifying relevant studies, we first conducted an automated search dated May 2023 of medical (PubMed, Embase, CINAHL, PsycINFO, and Cochrane), open-domain (Web of Science), engineering (IEEEXplore), and preprint (arXiv) academic articles databases using keywords from Table 1 generated during stage 1. Including preprint and engineering databases allows the search to extend to perspectives outside of the medical domain. The arXiv preprint archive was searched to account for more recent articles currently unavailable in peer-reviewed publication databases [47]. Duplicated articles and articles found in the preprint archives that were published will be removed from the pool of potential studies.

We undertook a pilot review on a manageable sample of the more relevant studies to refine the search strategy. The pilot review process allows us to refine our inclusion and exclusion criteria further. The pilot review team comprises at least one senior clinician and a senior data scientist with relevant health care domain experience to guide the construction of search strings—these were refined in a series of team discussions in consultation with a medical librarian. Multimedia Appendix 1 provides details of the source database-specific search strings used for each concept, as shown in Table 1. Differences in search strings are due to discrepancies on what databases can accommodate in a search (eg, wildcard characters may be adapted in some but not in others, and databases may vary in the type of subject heading indexing used). Two junior researchers (MD and YLC) collated the pool of articles identified from these search strings.

### Stage 3: Study Selection

A 2-step screening procedure was adopted here—a title-abstract screening followed by a full-text screening was conducted for the articles identified from stage 2. Two reviewers independently screened the articles using the criteria presented in the succeeding paragraphs. The articles’ titles and abstracts (ie, step 1 screening) and the full text (ie, step 2) were the basis for screening. The criteria for study selection are continuously revised through regular meetings. The reviewers arrange meetings to resolve any disagreements. The senior reviewers in the study team are consulted to reach a consensus when screening conflicts arise. According to the proposed guidelines for scoping reviews [48,49], we report the article search and screening results in a PRISMA-ScR (Preferred Reporting Items for Systematic Reviews and Meta-Analyses Extension for Scoping Reviews) [50] flow chart shown in the results section.

Our review included the following studies: (1) studies that directly support clinical decision-making specifically for diagnostic, triage, screening, prognostic, and prescriptive purposes; (2) studies that use AI, computer-executed algorithms, machine learning, and traditional statistical multivariate techniques; (3) risk prediction models for a disease condition or a future health outcome; (4) studies that assess CDS models in predeployment stage for its potential impact; (5) studies that propose the use of simulation-based optimization during model development; (6) human studies; (7) experimental or observational studies—including prospective, retrospective, and ambispective studies, clinical trials, pragmatic clinical trials, and validation studies; (8) studies that are published in journal articles, conference proceedings, and preprint archives; (9) studies written in English with no constraints on the year of publication; and (10) risk prediction models for a disease condition or a future health outcome.

We excluded the following studies: (1) studies that do not involve clinical domains as prediction outcomes; (2) studies that focused on the use of AI as therapy (eg, treatment monitoring and glucose control systems); (3) studies that use machine learning, pattern recognition, AI for descriptive analysis; (4) pathological specimen and sensing device signals accuracy-related studies; (5) image segmentation/registration only without classification/prediction with clinical end points; (6) studies that deal with purely system/population level outcomes that are irrelevant to patient-provider interactions; (7) pure qualitative evaluation for clinical usefulness; (8) purely methodological papers on medical data processing (eg, image processing and noise filtering) without specific application domain; (9) studies that use purely ex silico evaluation typically require either a partial or complete deployment of the developed CDS system (eg, randomized controlled trials for actual impact assessment); (10) studies that only use traditional metrics—for example, area under the receiver operating characteristic curve, area under the precision-recall curve, mean squared error, accuracy, sensitivity, specificity, goodness-of-fit, and other discrimination-related measures only—to validate CDS models, systems, and tools, that is, those studies that do not consider broader systems-level usefulness; (11) studies that do not report model development process (eg, proprietary CDS tools or systems) as these do not disclose sufficient information about underlying technology and algorithms; and (12) studies which report reviews (eg, scoping reviews, systematic reviews, and rapid reviews).

As our primary aim is to exhaustively review the published potential impact evaluation strategies done in silico for CDS, literature or scoping review studies were excluded. No other articles were excluded based on the year of publication. Deduplication was done using Zotero [51].

### Stage 4: Data Charting

#### Overview

Data charting will collect critical information to answer the research question for the articles extracted from stage 3 after the 2-step screening process. An a priori list of coding variables corresponding to this study’s concept framework and research questions has been developed (Table 2). Reporting [52,53] and data extraction [54] guidelines related to the concept framework guided the selection of coding variables to be extracted from the screened article database. As studies may or may not conform to these guidelines, and new categories and subcategories may be derived from the literature, the a priori coding variables may change. The emergent categories and subcategories will be checked for co-occurrences (overlapping concepts) and redundancies. Codes with the same concepts will be aggregated and refined to maximize mutual exclusivity and exhaustiveness. Aside from these structured coding variables, we shall also extract general information about the articles as guided by related published review protocols [55-57].

The data charting form will be developed in a shared collaborative Notion.so [58] database with the structure in Multimedia Appendix 1. The form was designed and maintained by an arbiter who ensures it is comprehensive and flexible. The charting process will be initially blinded. As with the article screening, we conducted a pilot charting trial to validate the encoding items. Each researcher can only see their respective chart to facilitate independent charting. An initial charting form is presented in Table 2. Team discussions were held as the team progressed in the charting process to consider other items to extract. At least 2 reviewers will be assigned to each article for validation. Any discrepancies will be resolved together with the entire team for the final determination of the charted codes. After the pilot trial, all articles included from stage 3 will undergo charting, resulting in an encoding database for this review.

**Table 2:** Data extraction items.

Data extraction broad concepts	Specific items extracted
Characteristics of the studies included	Publication year Research location (ie, country) Data source Data collection design Collection period Patient cohort description
Decision-making objectives and end points	Objective of the CDS^a^ model (ie, triage, diagnostic, prognostic, and prescriptive) Specific decision-making tasks assisted by CDS Clinical domains
In silico^b^ evaluation metrics	Specific evaluation metrics General themes of the metrics (ie, patient, process, provider, and cost-effectiveness outcomes)
In silico evaluation frameworks	Simulation modeling paradigm Simulation modeling objective Simulation parameters (parameters and parameter groups) Reported reusability Access to codes and tools used to conduct the simulation

^a^CDS: clinical decision support.

^b^Evaluations via computer simulations of clinical workflows during preimplementation.

#### Characteristics of the Studies

We include any study that reports the development of an expert system, a computer-aided clinical decision-making tool, or CDS with an underlying rule base or machine learning—including supervised, self-supervised, and unsupervised methods; deep learning; and reinforcement learning. We also include the more traditional multivariate analysis-based CDS such as linear, logistic, and Cox regression approaches to clinical scoring systems and prediction rules [38]. We encode the type of methods or algorithms used, their reported advantages (ie, aside from empirical performance reported), the disadvantages of the method, and their dependencies on data and the population from which the data was collected, as reported by the study authors. We also collected information about where the research was conducted and the year of publication.

#### Decision-Making Objectives and End Points

Since CDS model outcomes are directly related to its intended task and use, it is necessary to understand the scope of the desired outcomes from the predictions for an objective potential impact assessment. The development and use of clinical rules predate AI-based CDS. The outcomes of these clinical rules are broadly classified into diagnostic, prognostic, and prescriptive outcomes [52,54,59]. We adopt this same classification for AI-based CDS. Diagnostic outcomes generally predict the risk for a particular condition or disease (based on existing health data) to support early intervention or screening decisions. Prognostic outcomes indicate the future course of an illness or disease, including the likelihood of recovery, quality of life, complications, or mortality. Some CDS studies may prescribe treatment beyond diagnosis or prognosis end points [1,59]. Guidelines for reporting [52,53] and appraisal [54] prediction models mention a comparable taxonomy of CDS outcomes. Another reporting checklist for studies that use AI in medical imaging CDS differentiates the intended use (eg, diagnosis, screening, and staging) with the tools’ proposed role (eg, triage, replacement, or add-on) [60]. We shall consider these classifications in our analysis.

#### In Silico Evaluation Metrics

We explore methods to evaluate CDS’ potential impact in silico on clinical workflow operations, patient outcomes, and economic outcomes [5]. Our aims take inspiration from the renewed focus of health care towards the Quadruple Aims, which adds the well-being of care providers as a fourth dimension, in addition to the traditional aims of improved patient experience, better health of populations, and cost reduction [41,61]. This underscores the need to devise workflow-sensitive evaluation methods, for example, considering how CDS sustains service providers’ productivity (eg, referral rates as a process metric) within a resource-constrained care pathway. Further, we consider how reported studies propose the measurement of potential impact on patient health beyond the traditional accuracy-related measures [38], such as net benefit [62], realized [24] net benefit, and length of stay [63]. Some studies examine how implementing CDS systems impacts hospital budgets, with related metrics including costs and the incremental cost-effectiveness ratio [64]. More broadly, these metrics may be used to validate potential impact across different periods and study sites; this allows the monitoring of CDS performance consistency and the prompt triggering of model updates when necessary [15].

#### In Silico Evaluation Frameworks

Simulation modeling is a powerful tool for analyzing complex systems by creating representations that mimic the real world. It allows researchers or decision-makers to study how the system will behave over time prior to the actual deployment. Different simulation methods can be characterized by specific attributes, such as discrete or continuous, static or dynamic, and stochastic or deterministic [32]. A discrete simulation models the state of the system at distinct time points. For example, the number of patients in the waiting room only changes when a patient arrives. In contrast, a continuous simulation models the parameter that changes over time regardless of any triggers that change the state of the parameter. A static simulation models the system only at a specific time point, while a dynamic simulation studies the system’s evolution over time. Last, a stochastic simulation involves randomness where simulation parameters can be probabilistic. For example, the patient arrival times may follow a specific distribution. However, a deterministic simulation encompasses parameters that have specific values. For instance, if the simulation sets the number of patients per time interval to 10, the value of such a parameter will stay at 10 throughout the simulation.

Our study focuses on existing research that reports using workflow simulation methods to assess the potential impact before embarking on often challenging and costly actual impact assessment. In silico evaluation can provide a more robust basis for successful implementation trials. As such, we consider studies that evaluate AI tools through an in silico approach without the need for actual deployment. Strategies may use reinforcement learning that optimizes a policy for multiple stages of decision-making (ie, such as machine learning–assisted treatment selection) [65]. Another approach may model a clinical care pathway as a discrete set of states and transitions [25], namely, DES, a popular method in health care workflow simulation to study resource allocation as it incorporates how resources change according to triggering events [36]. Another method, ABM, is particularly useful for modeling the interactions between various entities (ie, health care workers) in a clinical workflow [11]. Studies may also use a retrospective evaluation using cross-validation and decision curve analysis [24,62,66] to measure a decision-analytic score. As data extraction proceeds, we shall consider the more precise taxonomy of the simulation modeling [32] while broadly accounting for other in silico approaches, such as the examples. Other paradigms used will be encoded and reported as they arise.

We further consider the intended purpose of simulation, that is, the simulation modeling objective, which may fall under 1 of the three initial categories: (1) to conduct a straightforward measurement of clinical usefulness metrics, (2) to analyze the sensitivity of outcomes to various workflow parameters and scenarios, and (3) to optimize decision-making capability of CDS via a care pathway simulation.

Last, we consider the parameters used to construct the in silico clinical workflow. Specifically, we shall evaluate how patient, provider, process, and cost considerations are represented as simulation parameters. These clinical workflow factors describe the real-world care pathway, including patient condition states, treatment or intervention events, resource availability, duration of events, and many other factors.

### Stage 5: Collection, Summarizing, and Reporting of Results

We will collect the data in a table of values corresponding to each variable (ie, column) and each relevant article (ie, row). An analysis of the values extracted will be done to identify sparse- and well-studied themes within and across critical concepts. Frequency and thematic analysis will be used for this analysis [44,67]. Themes combined with the extracted textual information will allow for the study of trends. Univariate and multivariate statistics will be reported as deemed relevant for each type of analysis. Descriptive statistics and charts will be used to describe the characteristics of the included study across the variables listed in Table 2. When appropriate, ANOVA, Kruskal-Wallis, and Pearson chi-square tests will compare trends across different categories. The association of variables based on co-occurrence will also be investigated. Further, the reusability of any software artifacts or code repositories associated with included studies will be reported according to the claims of the articles’ respective authors. The reporting of results will follow the PRISMA-ScR guidelines [50].

### Stage 6: Dissemination and Stakeholder Consultation

Beyond summarizing the results and findings, we will consider the overall implications of the findings for the in silico potential impact assessment of algorithm-based CDS systems, models, and tools. This scoping review will support the development of a draft framework that will guide clinical workflow simulation modeling for impact assessment, with specific considerations on the model purpose, evaluation scope, objectives, and strategy. This framework will further support the in silico evaluation of proposed CDS studies collected through discussion with potential stakeholders—implementation scientists, modelers, and clinicians conducting. This will also allow stakeholders to provide a higher level of interpretation, domain expertise, and perspective to validate the findings further and support effective knowledge transfer and uptake of evidence to ensure the usefulness of the scoping studies for AI developers and clinical researchers [43].

## Results

Our review began with an automated search of selected databases in May 2023. The resulting articles were managed using Zotero [51] and Notion.so [58] for automated article metadata collection and note-taking, respectively. Most of the title and abstract screening were finished by November 2023. However, the review team allows for flexibility as the screening criteria are refined throughout the review. Full-text screening proceeded from December 2023 to May 2024, including hand-searching and reference chaining. Charting was concurrently done with the full-text screening. Analysis and writing of the full scoping review results will be finalized in July 2024. The reporting of this scoping review protocol and results as published literature will be from July 2024 to February 2025.

**Figure 1:**
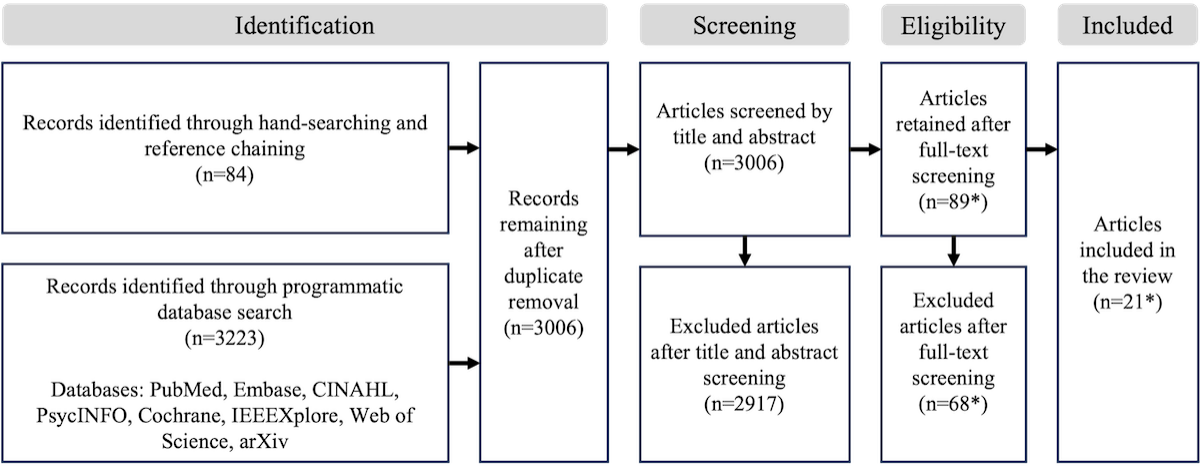
PRISMA-ScR (Preferred Reporting Items for Systematic Reviews and Meta-Analyses Extension for Scoping Reviews) flowchart. Asterisk (*) indicates data as of April 2024.

The current stage of our scoping review yielded the results shown in Figure 1. At the first screening stage, most articles were excluded based on titles and abstracts that did not fulfill the inclusion criteria. Moreover, we also excluded at this stage studies that suggest the development of CDS tools but only perform an evaluation using the area under the precision-recall and receiver operating characteristic curves, accuracy, precision, recall, *F*_1_-score, and other traditional confusion matrix-based scores; further, these were studies that do not attempt to evaluate potential impact and usefulness to its intended clinical care pathway placement [38]. Additional studies were also excluded due to the focus on algorithmic developments in processing medical data (ie, image, text, and structured data). On the contrary, articles that mention usefulness and impact evaluation without providing further details in the title and abstracts were included in the full-text screening.

In the second stage of the detailed full-text screening (ie, using the full text as the basis), more articles were excluded due to the lack of potential impact and usefulness evaluation. Other articles were excluded as duplicates not detected in the initial automated deduplication based on article metadata. A few articles were also excluded due to unavailable full text, as only extended abstracts were published. As this stage is ongoing and considering that reference chaining may still be conducted based on the screened articles, the final number of relevant articles will be reported along with the scoping review results. As of April 2024, 21 articles are included in the review.

## Discussion

### Principal Findings

The proliferation of AI models in health care encourages researchers, patients, and providers to use these technologies to optimize the care delivery processes. Yet, only some models are being translated into clinical practice [6]. The ability of data-driven machine learning methods to generalize to different temporal and geographical patient cohorts is challenged by often changing real-world medical data [17]. This demands more robust and adaptive approaches to encourage user acceptance and trust [12,13,16]. Traditional impact assessments based on pilot implementation for health technology assessment can be resource-intensive with the rapid proliferation of new models [68,69]. The in silico evaluation of algorithm-based CDS provides a resource-efficient framework for estimating novel CDS’ potential clinical impact to facilitate the seamless integration of a model into the workflow. Moreover, computer simulations require much fewer resources and have less direct implications for ongoing patient care, allowing for regular and repetitive use throughout the CDS development and maintenance lifecycle. Our review aims to analyze and report the scope of using in silico CDS evaluation in published academic literature. We expect that the results will uncover the clinical decision-making domains where such evaluations are used or, otherwise, underused, how clinical workflows are simulated, the potential impact metrics used to illustrate the usefulness of CDS, and areas where more research is necessary.

Several US Food and Drug Administration–accepted patient simulators [70,71] and other approaches [30,68] that simulate patient characteristics enable an in silico evaluation of patient-level impact and have been proposed to be used at a preclinical stage. These simulators allow the assessment of the response to different treatments by the same patient—which is unlikely in real-world treatment scenarios due to dynamic patient conditions. Comparably, clinical workflow enables simultaneous evaluation of various scenarios using the same patient cohort characteristics, sharing the same validation capabilities and objectives [72,73]. However, clinical workflow simulations encompass a broader perspective, considering the efficiency and effectiveness of treatments and interventions, such as CDS, across the entire care pathway.

In a preliminary collection of included articles, the broader perspective was demonstrated by the accounting for process-related factors. For example, time intervals (ie, door-to-doctor time) between workflow events were considered by Alenany and Cadi [63] as an evaluation metric. Misic et al [23] and Rodriguez et al [74] focused on patient volume and referral rates to evaluate workflow throughput. In addition, other studies simultaneously assess patient outcomes with cost-related outcomes, such as length-of-stay and costs per visit [75], early-stage cancer detection rate and cost savings [76], and intensive care unit length-of-stay and corresponding costs [77]. Yin et al [5] highlighted that in the real-life evaluation of AI applications, the outcomes considered can be grouped into patient, cost-effectiveness, and clinician outcomes. We consider this in our review and propose distinguishing between provider, that is, clinician outcomes and process outcomes [22], expanding the outcome themes into 4 categories.

Furthermore, similar themes can also be applied to categorize clinical workflow factors, that is, parameters, used in the design of an in silico care pathway. Lee et al [35] considered time intervals between events as simulation parameters. Other studies [23,25] used provider-related parameters such as provider effectiveness and carrying capacity. Other studies simultaneously consider patient-, cost-, and process-related outcomes—such as in [35,77]. These parameters are typically based on historically observed data distributions, expert judgment, cited from published literature, or determined from prospective time-motion studies. We will report such a basis for parameter initialization accordingly.

The interplay of multiple outcomes and drivers and the expansion of health care also aim to consider provider well-being, which adds to the complexity of impact evaluation. Different simulation modeling paradigms are proposed to handle such complexity—such as in the DES frameworks used by [25], the ABM done by [35], and state-transition models shown in the microsimulation by Rodriguez et al [74], and in the Markov-based transition model used in [78] evaluating wait time–saving effectiveness of an AI-based CDS. Moreover, dynamic treatment regime optimization is proposed to capture staged treatment scenarios and optimize outcomes [65]. Last, considering cost-effectiveness approaches, decision trees can also be used to capture costs and benefits attributed to a hierarchical decision-making scenario; this is demonstrated by Tsai et al [77] to evaluate an extubation failure prediction CDS.

While extensive literature on the usefulness of simulation modeling and knowledge of the simulation of clinical care pathways as avenues for CDS in silico evaluation still lack consolidation [79], possibly due to the significant context dependencies across different health systems. When translated into a quantitative modeling framework for rigorous, objective evaluation, the diversity, human-centricity, and complexity of clinical workflows pose unique challenges [18]. Addressing these challenges requires interdisciplinary groups familiar with hospital management, clinical context, process nuances, and the availability of necessary modeling capabilities. Despite the prevalence in the reported development and advancements of CDS models, accelerated by the surge in AI methods—studies that reported the extent of clinical and workflow impact through in silico evaluation are still relatively sparse [5,6]. A critical need remains to reassess the current model simulation practices to advance this field. We believe this will expedite the integration of novel CDS system development. To the best of our knowledge, this is the first review that aims to understand CDS system in silico evaluation methods beyond traditional accuracy metrics.

### Conclusion

This scoping review follows the framework proposed by Arksey and O’Malley [42] and other recent enhancements [43-45]. We searched 8 medical-focused and general academic domain databases to gather articles from an interdisciplinary perspective. An automated search followed by a 2-step screening process was done to implement the scope of the review. Unlike previous reviews, we will specifically include CDS related to traditional multivariate models and machine learning. In addition, we designed a data charting table based on discussions with the multidisciplinary review team and previous reviews on related topics. This table will guide the data extraction phase, and the items will be flexibly revised along with further study of the included articles. Finally, we plan to summarize our results using descriptive and co-occurrence analyses. For example, the distribution of race and ethnicity of collected patient information—as reported by the included articles—will show how fairness is represented in current AI research in health care. Similarly, an analysis of co-occurring themes (ie, in statistical analysis methods, CDS decision-making tasks, evaluation metrics, and simulation paradigms) may surface clinical domain-specific and domain-agnostic approaches to in silico potential impact evaluation.

We anticipate our results will be informative about the state-of-the-art in silico evaluation method based on workflow simulation models and the associated outcome metrics and targets. More specifically, our results will describe the characteristics of the clinical decision-making domains being modeled, the relevant measures of impact that are simulated, and how such are captured in clinical workflow simulation. As we also aim to report the reusability of methods cited, our work will serve as a springboard for the reader to find suitable in silico evaluation frameworks, software artifacts, and code repositories. Ultimately, our work is a starting point in developing a unified in silico evaluation framework adaptable to various clinical workflow scenarios.

### Limitations

There are several limitations to our approach. First, while some guidelines for reporting may exist, they may need to be revised to cover the variety of studies in our criteria. For example, a transparent reporting of machine learning models developed for diagnosis, prognosis, or prescriptive analytical support is still being developed [27], and conformance to these guidelines may influence the extent and precision of our data charting. Second, a critical appraisal of articles will not be done as we primarily aim to provide an overview of the scope by which in silico evaluation methods have been used. Third, we also included reports from e-Print archives (arXiv), trading off a more exhaustive scope versus the inclusion of non–peer-reviewed articles; an accounting of such articles will be provided in the reporting results. Last, we included only English articles; thus, we cannot extrapolate our findings to publications in different languages. Our findings will add to the knowledge of applications of statistical learning and simulation methods in health care.

## Appendix

Multimedia Appendix 1 Supplementary material showing the keywords used and other details of the literature search, including a sample encoding sheet that uses Notion.so.

## Abbreviations

ABM: agent-based models
AI: artificial intelligence
CDS: clinical decision support
DES: discrete events simulation
PRISMA-ScR: Preferred Reporting Items for Systematic Reviews and Meta-Analyses Extension for Scoping Reviews

## Data Availability

All data generated or analyzed during this study are included in this published article and its Multimedia Appendix files.
